# Dr. William Butchart and "The Milne Treatment."

**Published:** 1912-07-20

**Authors:** 


### DR. WILLIAM BUTCHART AND "THE MILNE TREATMENT."

### Honour for a Scotch Superintendent.

A public presentation has been made to Dr. William Butchart, till lately Medical Superintendent of the Blawarthill Hospital of the Renfrew and Clydebank Joint Hospital Board. The ceremony took place in the Burgh Court Room, which was crowded with Dr. Butchart's supporters, and Mr. John Hutcheon, who presided, said :

Dr. Butchart, as they all knew, refused to sacrifice his principles to position in the matter of a certain treatment of scarlet fever, and thus a long and honourable connection of fifteen years, in which his work had never been discredited, was severed. While admiring him for his independence, carried out at great sacrifice to himself, they were mainly there to honour him as a man who had long occupied a prominent position in Clydebank. Both as a County Councillor and as a member of the School Board he did excellent work. He referred particularly to his services in the promotion and teaching of ambulance classes. What had been contributed was the spontaneous offering of those who know him and respected him, and it was the wish of the Committee, as it was of himself, that he would have the satisfaction of knowing that his services to the town had been recognised to some extent.

Mr. Hugh Miller then made the presentation.

Dr. Butchart, in the course of his reply, referred to his work in the hospital during the past fifteen years. An epidemic, he said, struck the town, and then there came a period of scientific investigation which culminated in some of their leading men discovering that there was a new method of treating scarlet fever—a method which, the doctor remarked, was as old as the hills—and that system of treatment was adopted for their children. He did not wish to ascribe fault to anybody, but there seemed to be a big blunder made somewhere, especially when the men whom they sent to their public boards to do preventive work were beginning to do curative work. Prevention should have been their whole object, but that was apparently forgotten. He wanted them to understand the position a little bit. They were at the tail-end of an epidemic which struck Clydebank somewhat forcibly. The work did not cost more than any other place, although he knew some of their friends discovered that the cost per patient was a long way more than anywhere else. As a matter of fact, it was a long way less than any place else, and the results, he thought, were fairly good when they got a death-rate of .5 per cent. in scarlet fever. At the time the epidemic was nearly at an end the curative process began, but it was a process of intense cultivation which spread the disease all over the district, and they got grand results. They were led to believe there was a great diminution in the cost per patient; but he did not think it had reduced their taxes or was likely to do it in the end. In the month of September, when they got the financial statement, they would find that the cost per patient of the Milne treatment would work out at something like £8 or £8 8s. per patient, whereas in the previous year it was £6 10s. when he had the responsibility. If the Milne treatment was to diminish the cost and save the taxes well and good; but was there a man in Clydebank who refused or grumbled at paying his public health taxes? The children were removed for the sake of the general public and for their own sakes. He was not out to save the ratepayers' money, because he knew the ratepayers trusted their children to him, and that trust had been honoured in every sense. The outcry about saving the ratepayers' money was simply a bogey.

Dr. Butchart then gave his reasons why he refused to have anything to do with the Milne treatment. He knew what the treatment was. He was at the hospital to do his best for the patients, give them the best and latest medical skill that could be adopted, and Blawarthill, he ventured to say, was ten years ahead of anything in Scotland when he left it. He had been appointed superintendent, and was responsible for the lives of the patients entrusted to his care. He was not there to experiment on their children, but to get them better and help to diminish the spread of the disease in the locality. His duty began with curative work and ended by helping in the preventive work of the district. When that matter came up he declined to risk his professional reputation, because he knew what the Milne treatment was from practical experience. He thought by doing so he would cause others to hold their hands, but they did not. He denied that the Hospital Board was responsible for the treatment of the patients; and if anything went wrong through carelessness or negligence an action would have been raised, not against the Board, but against the individual. That being so, the Board wanted to experiment at his expense. He was to take the risk, and they were to save so much money to the ratepayers. Even if he did not consider it a foolish experiment, was it fair to ask that it should be done, and was it fair to say that the Board were responsible? On the Board he had some very good friends, including Bailie Hogg and Councillor Mann, and they with others realised the importance of the position and fought manfully all through, but were unsuccessful. When they dismissed him they took some part of his living from him, which they had done their level best to ameliorate. He paid a tribute to the splendid help he had received from his wife during those trying times, and, in conclusion, on her and his own behalf he thanked all most sincerely for their splendid gifts.

Accompanying the address, which was signed "John Hutcheon, Chairman, Daniel M'Kinlay, Secretary, Hugh Miller, Treasurer," was a testimonial, signed by all the Committee, as follows :—

"The Committee of the public testimonial presented at this date consider it their duty to inform you that in presenting same they have embraced the opportunity brought about by your severance with the Renfrew and Clydebank Joint Hospital. It is in our knowledge that a large and intelligent section of the community deprecate the manner in which certain members of the Hospital Board interfered between you in your professional capacity and the treatment of the patients, and these citizens plainly express their admiration of the manner in which you stood for the interests of the community on your professional interpretation of the position even to your own loss. We assure you of the esteem in which you are held, and of the undoubted faith in your professional judgment so far as we can gather from the high rate of recovery of the patients entrusted to your care at the Renfrew and Clydebank Joint Hospital, and the exceptionally low death-rate you have always maintained there."

Mrs. Butchart was also presented with a silver salver.

